# Dynamic graph convolutional networks with attention mechanism for rumor detection on social media

**DOI:** 10.1371/journal.pone.0256039

**Published:** 2021-08-18

**Authors:** Jiho Choi, Taewook Ko, Younhyuk Choi, Hyungho Byun, Chong-kwon Kim

**Affiliations:** Department of Computer Science and Engineering, Seoul National University, Seoul, Republic of Korea; Fuzhou University, CHINA

## Abstract

Social media has become an ideal platform for the propagation of rumors, fake news, and misinformation. Rumors on social media not only mislead online users but also affect the real world immensely. Thus, detecting the rumors and preventing their spread became an essential task. Some of the recent deep learning-based rumor detection methods, such as Bi-Directional Graph Convolutional Networks (Bi-GCN), represent rumor using the completed stage of the rumor diffusion and try to learn the structural information from it. However, these methods are limited to represent rumor propagation as a static graph, which isn’t optimal for capturing the dynamic information of the rumors. In this study, we propose novel graph convolutional networks with attention mechanisms, named *Dynamic GCN*, for rumor detection. We first represent rumor posts with their responsive posts as dynamic graphs. The temporal information is used to generate a sequence of graph snapshots. The representation learning on graph snapshots with attention mechanism captures both structural and temporal information of rumor spreads. The conducted experiments on three real-world datasets demonstrate the superiority of Dynamic GCN over the state-of-the-art methods in the rumor detection task.

## 1 Introduction

Social media has been a great disseminator for new information and thoughts. Due to its accessibility of sharing information, however, social media has also become an ideal platform for propagations of rumors, fake news, and misinformation [1]. Although the definition of rumor may vary by literature, we use the term rumor to indicate messages in which the veracity labels are unknown at the time of diffusion [2, 3]. Rumors on social media not only mislead the users of online but also affect the real world immensely [4]. Thus detecting the rumors and preventing their spread became an essential task.

Early studies in rumor detection focused on understanding the characteristics of rumors [5, 6] and extracting prominent features of rumor from the textual contents or the users’ profiles [7–11]. Also, the temporal features or propagation patterns were elaborated significantly in [12–17], respectively. These elaborated features show profound results in rumor detection tasks. The manually extracted content-based, user-based, or propagation-based handcrafted features were used to train classical machine learning classifiers such as a decision tree, random forest, or SVMs. However, the limitation of using manually extracted features is that it fails to capture the high-dimensional patterns of rumors.

To solve the problem of using handcrafted features and avoid the feature engineering efforts [18–21], had adopted neural networks such as recurrent neural networks (RNNs) or convolutional neural networks (CNNs). The proposed rumor detection models were able to capture the high-dimensional representation from the textural contents, user profiles, and propagation structures. The models of using the propagation structure [20, 21] try to represent the skeptical or conflict opinions of the responsive posts such as retweets, replies, or comments toward the original message.

Recent advent in Graph Neural Networks (GNNs) and its variants such as Graph Convolutional Networks (GCN), GraphSAGE, and Graph Attention Networks (GAT) [22–25] have gained a lot of attention. The GNNs have shown promising results in graph inference tasks such as node classification, graph classification, and link prediction. [26, 27] successfully adopted GCN and GAT in the rumor detection domain, respectively. However, both models aren’t considering the temporal dynamics of the rumor propagation, which only considers the static graph structure of the final state of rumor propagation.

**Fig 1:**
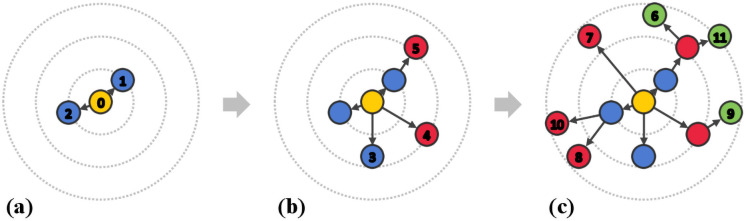
Example of an evolving rumor propagation network where each node represents a post. The link between nodes implies a direct reaction. The numbers in nodes are in the chronological ordering of the generation, and the distance from the root post represents the time interval. The colors depict users’ latent stances on their parent’s post (e.g., neutral (blue), suspect/doubt (red), trust/support (green)). Example scenario: there is an initial claim of Node 0. Node 1, 2, and 3 express neutral stances to the initial claim. Node 4, 5, 7, 8, and 10 express suspect/doubt and Node 6, 9, 11 express trust/support on their parent posts, respectively. Can we identify the veracity label of Node 0?

In this study, motivated by the dynamic nature of rumor propagation, we present a novel graph convolutional network-based model, named *Dynamic GCN*, to better understand the evolving pattern of rumor propagation. The model includes two distinct ways of representing rumor propagation with graph snapshots: sequential and temporal snapshots. Fig 1 depicts how the rumor propagation can be represented with the sequence of snapshots. In the example scenario, the initial trust (Fig 1a) of the root post begins to gain doubts (Fig 1b), and the posts that reveal doubts are supported by others (Fig 1c). With this whole process, the veracity value of the root post can be inferred. The details of the representation will be discussed in section 4. The extended GCNs capture the spatial representation of rumor posts within a snapshot. And finally, the series of graph snapshot representations are combined with an attention mechanism. We evaluate the proposed model with three real-world datasets and show our model outperforms other state-of-the-art methods.

We summarize the main contributions as follows:

- We propose two distinct ways of depicting a dynamic graph by generating two variants of graph snapshots: sequential and temporal snapshots.
- We propose a novel GCN-based rumor detection model that can capture the evolving pattern of rumor propagation by aggregating the structural representations of snapshot sequences.
- The conducted experiments on three real-world datasets demonstrate that our model accomplishes superior results on the rumor detection task compared to other state-of-the-art methods.

We organize this paper as follows. In Section 2, we briefly review the rumor detection methods and the fundamental components of our model; GCNs and attention mechanisms. In Section 3, we formulate the rumor detection problem with the propagation structure of rumor. In Section 4, we introduce our model as follows: snapshot generation, graph convolution networks, readout layer, attention mechanisms, and prediction. In Section 5, the details of experiments and performance evaluation are described. And finally, we conclude this work in Section 6.

## 2 Related work

### 2.1 Rumor detection

Rumor is commonly defined as a message in which the veracity labels are unknown [2, 3]. Rumor detection on social media is a task of classifying messages or posts with their veracity labels. Traditional approaches in rumor detection and other misinformation detection are to extract handcrafted features with prior knowledge on rumors. The content-based method and user-based method were two main approaches [7–9, 11]. To elaborate different and additional features, the temporal or linguistic features were considered in [12–14]. Another characteristic feature of the rumor is its propagation structure. [15–17] utilize propagation patterns of rumor and show profound results on rumor detection. The manually extracted content-based, user-based, temporal, or propagation-based handcrafted features were used to train classical machine learning classifiers such as a decision tree, random forest, or SVMs. However, the limitation of models with handcrafted features is that they fail to capture the high-dimensional patterns of rumors. To solve the problem [18, 19], adopted deep learning models such as RNNs or CNNs variants to extract texture, image features, or user profile features from the rumor posts. Noticeably, models which utilize propagation structure as additional features that try to represent the skeptical or conflict opinions from the responsive posts. Recently, sophisticated models like GCN [26] or GAT [27] have successfully been adopted in the rumor detection domain.

### 2.2 Representation learning on graphs

Promising results on neural networks in various fields, encourage studies to bring deep learning to topological graph structures. Early studies of node embedding [28, 29] leverage sampling method like random walk for shallow node embedding. Recent advent in graph neural networks (GNNs) and its variants [22–25] made representation learning to be applied directly to a variety of graph structures such as social networks (friendship network, citation network, transaction network), knowledge graphs, computer networks, biochemical graph, and so on. One of the early and honored studies of GNNs is graph convolutional networks (GCNs) [23]. It approximates spectral filters with Chebyshev polynomial to extend convolutional operations on graphs. Another important study of the GNNs variant is GraphSAGE [24], which proposes different trainable aggregation functions from neighbor node embeddings with sampling methods. The proposed aggregation functions like mean, LSTM (random ordered), max-pooling are symmetric, where the ordering of neighbor nodes can be invariant. GAT [25] utilizes the attention mechanism for neighbor node embeddings. The GNNs have firmly established state-of-the-art performance in various graph inference tasks such as node classification, graph classification, link prediction, and community detection (clustering for the network structure). The fundamental component of GNNs is message passing architecture where the representation of the node is aggregated with its neighbors. The key differences in GNN variants are diverse neighborhood aggregation methods and different pooling approaches [30, 31].

### 2.3 Attention mechanism

The attention mechanism captures the importance of the input sequence by calculating the attention scores and weights. Compared to RNN-variants, such as Long Short-Term Memory (LSTM) [32], Gated Recurrent Units (GRU) [33], or Seq2Seq model [34], attention mechanisms have demonstrated outstanding results on both the efficiency and the performance in a variety of fields [35, 36]. Various attention mechanisms have been proposed depending on how they calculate the attention weights. [36] proposed additive attention, which adopts a feedforward neural network to calculate the importance of the input in the context of the input sequence. [35, 37] suggested dot-product attention and self-attention, which utilized dot-product similarity to capture the significance of certain input words from the set of words in the task of neural machine translation. Attention mechanism had also introduced and shown promising results in graph representation learning [25] where the node embeddings are calculated and attended over their neighbor nodes’ features.

### 2.4 Representation learning on dynamic graph

Graph structure like social network contains the property of dynamics by its nature [38]. Different approaches have been proposed to capture the dynamics of graphs. Early studies [39, 40] have focused on the changes or graph properties such as clusters, centralities, and similarities in certain temporal points of graphs called graph snapshots. From the advancement of feature-based dynamic graph representations, architectures with triadic closure and RNNs [41, 42] were adopted to embed sequences of graph structures. [43] suggested Dyngem which utilizes the snapshot method with an autoencoder to embed the evolving graphs. As the GNN-based methods have shown promising results on graph embedding tasks [44, 45], proposed GCN architectures combined with LSTM, GRU for the dynamic graph embedding. [46] applied a self-attention mechanism for representing the dynamic graphs.

## 3 Problem definition

In this section, the rumor detection task on graph structure is described. Rumor detection aims to predict the veracity label of a message. We formulate the task as below.

Let *C* = {*c*_1_, *c*_2_, ⋯, *c*_*m*_} be the set of *m* claims, where each claim (or a conversational thread) *c*_*i*_ consists of *n*_*i*_ microblog posts $P_{i}=\{p_{i0},p_{i1},\cdots ,p_{i(n_{i}-1)}\}$. The *p*_*i*0_ is the root post of *c*_*i*_ and *n*_*i*_ − 1 responsive posts are in chronological order by their post time. Each post *p*_*ij*_ is represented with *F* dimensional feature $x_{ij}\in R^{F}$.

Propagated from the root post, responsive posts form a propagation tree *G*_*i*_ = 〈*V*_*i*_, *E*_*i*_〉, where each edge represents its direct responsiveness [15, 16]. The vertex set *V*_*i*_ is represented with the posts’ features $\left\{x_{i0},x_{i1},\cdots ,x_{i(n_{i}-1)}\right\}$ and the edge set *E*_*i*_ represents set of directed edges from source posts (root or responsive posts) to their direct responsive posts. *A*_*i*_ is an adjacency matrix for the directed graph *G*_*i*_ and $X_{i}={\left\{x_{i0}^{T};x_{i1}^{T};\cdots ;x_{i(n_{i}-1)}^{T}\right\}}^{T}\in R^{n_{i}\times F}$ is the feature matrix for posts *P*_*i*_. Upon representing the propagation tree as a static graph, to elaborate its evolving pattern, we define the diffusion graph with *T* step series of snapshots $S_{i}=\left\{S_{i}^{\left(1\right)},S_{i}^{\left(2\right)},\cdots ,S_{i}^{\left(T\right)}\right\}$. The detail of the snapshot formulation will be discussed at section 4.1.

Each claim *c*_*i*_ is associated with its veracity label *y*_*i*_, where *y*_*i*_ belong to one of four classes {*T*, *F*, *U*, *N*} (True rumor, False rumor, Unverified rumor, or Non-rumor) or two classes {*R*, *N*} (Rumor, Non-rumor) depending on the dataset [16, 18]. The definition of rumor labels that we borrowed is the messages in which the veracity labels are unknown at the stage of the propagation and later classified by human annotators as true, false, or unknown. (non-rumor messages are thoughts or simple admiration) [2, 3]. In this study, we define the task of rumor detection as a supervised graph classification problem, which the goal is to learn a mapping function *f*: *C* → *Y* to classify the veracity labels of *c*_*i*_ using *S*_*i*_ and *X*_*i*_.

## 4 Dynamic GCN

**Fig 2:**
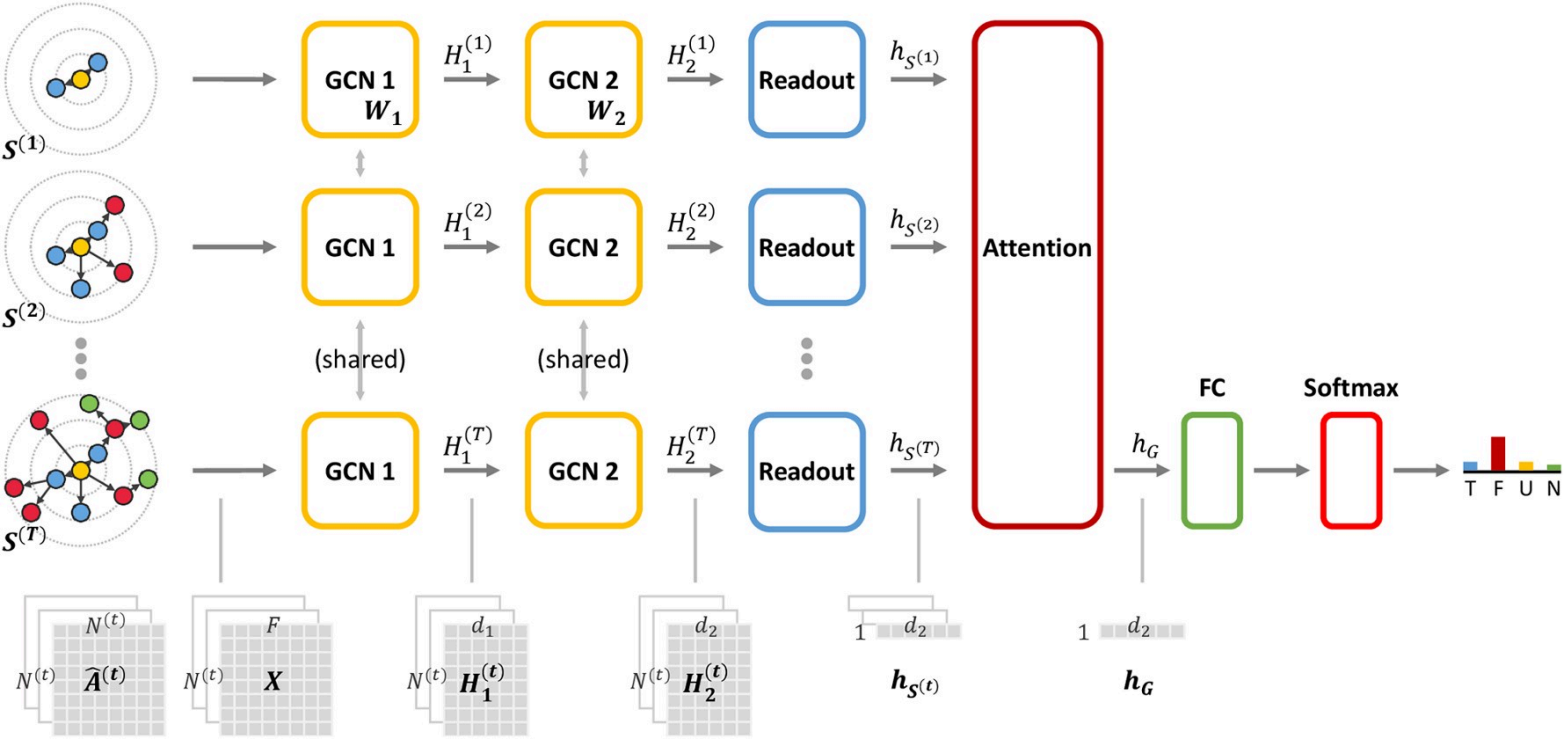
The overall architecture of Dynamic GCN rumor detection model with its layers and inputs’ dimensions for each layer.

In this section, we propose a dynamic graph representation learning model for rumor detection, named *Dynamic GCN* (DynGCN). The main components of the model are snapshot generation, graph convolutional networks, readout layer, and attention mechanisms. The components are respectively responsible for the following functionalities: rumor propagation representation, representation learning on a graph snapshot, node embedding aggregation for global graph representation, and sequential learning from the series of graph snapshots. Fig 2 is the overview of our dynamic rumor detection model with its layers and inputs’ shapes.

### 4.1 Snapshot generation

**Fig 3:**
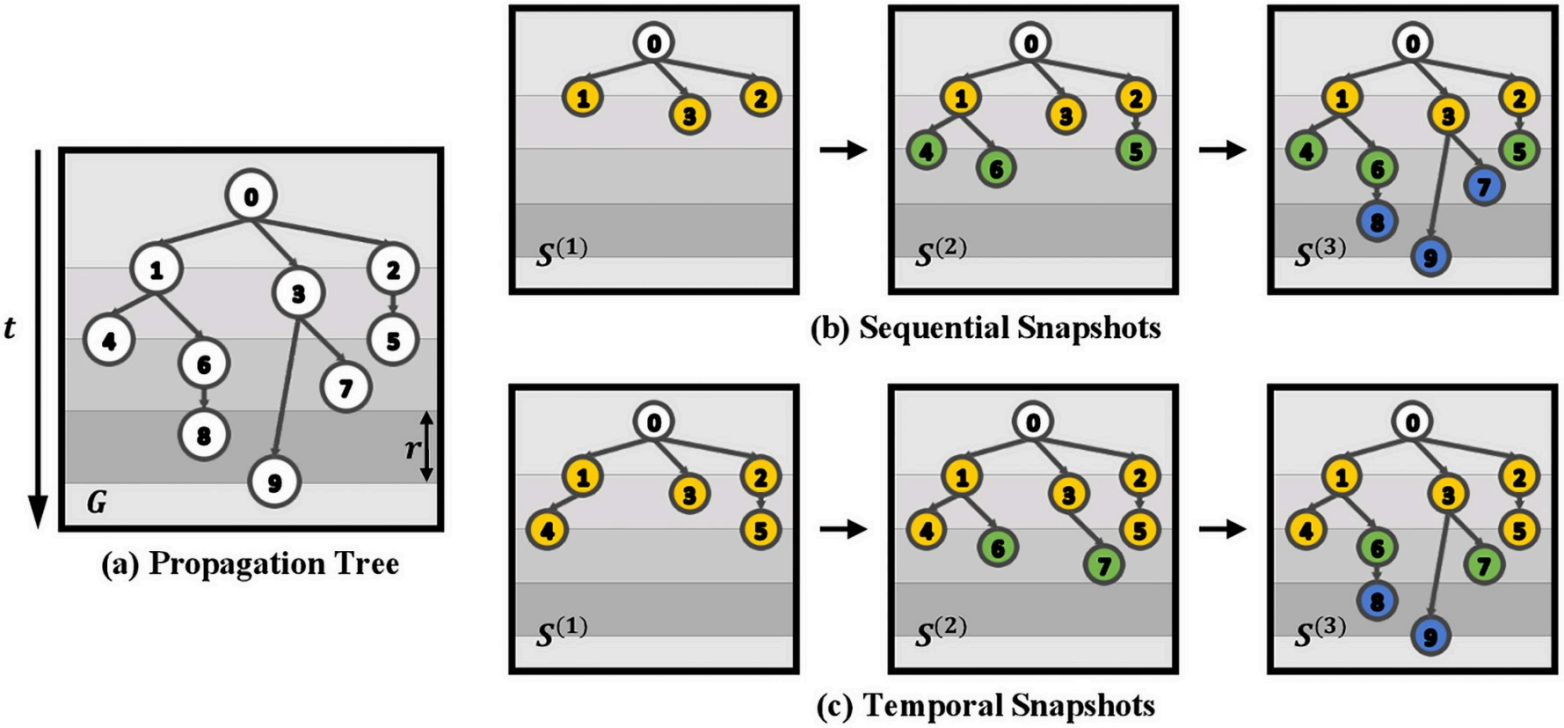
The disparity of snapshot generation results in sequential snapshots (b) and temporal snapshots (c) originated from the propagation tree (a). Sequential snapshots take account of chronological ordering and node counts, while temporal snapshots utilize timestamps.

To capture the evolving pattern of the rumor diffusion, we adopt the series of graph snapshots. We introduce two different ways of depicting the dynamic graphs as *T* step graph snapshots *S* = {*S*^(1)^, *S*^(2)^, ⋯, *S*^(*T*)^}. One is with sequential snapshots, and the other is with temporal snapshots. In Fig 3, we illustrated the two different methods of snapshot generations. Here on the index *i* for the claim *c*_*i*_ will be omitted. *S*^(*t*)^ is the graph snapshot at the time step *t*. Each graph snapshot in *S* will have separate adjacency matrices *A* = {*A*^(1)^, *A*^(2)^, ⋯, *A*^(*T*)^} with *S*^(*t*)^ = 〈*V*^(*t*)^, *E*^(*t*)^〉.

#### 4.1.1 Sequential snapshots

Consider the ordering of the additional nodes and links of the propagation tree. Starting from *S*^(1)^, the following graph snapshots will contain ⌈(*n* − 1)/*T*⌉ additional links (and nodes), where *n* − 1 is the total number of responsive links. Eventually, each graph snapshot *S*^(*t*)^ will contain ⌈*t* × (*n* − 1)/*T*⌉ links. The edge set for the sequential snapshot is as:

$$\begin{array}{c} E^{\left(t\right)}=\left\{e_{1},e_{2},\cdots ,e_{\left\lceil t\times (n-1)/T\right\rceil }\right\} \end{array}$$ (1)

#### 4.1.2 Temporal snapshots

Consider temporal information of the propagation tree. Compared to the sequential snapshot which contains the equal counts of additional edges, temporal snapshots separate *T* step diffusion with the fixed time interval *r*. Time interval *r* is retrieved by dividing the time difference of the first and the last responsive posts with the time step *T*. The edge set for the temporal snapshot *S*^(*t*)^ can be defined as:

$$\begin{array}{c} E^{\left(t\right)}=\{e|\tau_{e}-\tau_{e_{1}}\leq r\times (t),e\in E\},\\ r=\frac{\tau_{e_{\left(n-1\right)}}-\tau_{e_{1}}}{T} \end{array}$$ (2)

where *τ*_*e*_ is the timestamp of link *e*, and *r* is the time interval of the snapshots.

### 4.2 Graph convolutional networks

For the snapshot representation learning, we adopt graph convolutional architecture. Upon generating the graph snapshots *S* = {*S*^(1)^, *S*^(2)^, ⋯, *S*^(*T*)^} and their adjacency matrices *A* = {*A*^(1)^, *A*^(2)^, ⋯, *A*^(*T*)^}, we conduct representation learning on the graph snapshots with the graph convolutional networks (GCNs) [23]. Introduced in [23], the approximated normalized graph Laplacian [47] is used for high-dimensional node representation learning. Together with an adjacency matrix $A^{\left(t\right)}\in R^{N^{\left(t\right)}\times N^{\left(t\right)}}$, where *N*^(*t*)^ is the number of nodes in the snapshot, and feature matrix $X\in R^{N^{\left(t\right)}\times F}$, the learnable parameters $W_{k}\in R^{d_{k-1}\times d_{k}}$ are trained, where *k*^*th*^ layer produce node embeddings $H_{k}\in R^{N^{\left(t\right)}\times d_{k}}$. The GCN model that we adopted is as:

$$\begin{array}{c} H_{k}=\sigma (\hat{A}H_{k-1}W_{k}),\\ \hat{A}={\hat{D}}^{-1/2}\tilde{A}{\hat{D}}^{-1/2},\tilde{A}=A+I_{N},{\tilde{D}}_{ii}=\sum_{j}{\tilde{A}}_{jj} \end{array}$$ (3)

Trainable parameters W_*_ are shared between same level of GCNs with different snapshots steps. We use 2-layer GCNs with ReLU as activation function *σ*. We also adopt a skip-connection-like method [48] called feature enhancement [26] to enhance the information from a certain node, in this case, the root node. The root representations in a previous GCN layer bypass the layer as:

$${\tilde{H}}_{k}=concat(H_{k},{\left(H_{k-1}\right)}_{root})$$ (4)

And finally, inspired and introduced by [26, 49], instead of perceiving diffusion pattern as undirected graph, we adopt bi-directional GCNs which consider both direction of graph representation separately as:

$$H_{k}=concat({\vec{\tilde{H}}}_{k},{\overset{\leftarrow }{\tilde{H}}}_{k})$$ (5)

The outputs $H_{K}^{\left(t\right)}$, produced by the last layer *K* of GCNs, are node embeddings of each graph snapshot *S*^(*t*)^.

### 4.3 Readout layer

After the GCN layers embed node representation $H_{K}^{\left(t\right)}\in R^{N^{\left(t\right)}\times d_{K}}$ of each graph snapshot *S*^(*t*)^, the global graph pooling method is used to convert node representation to graph representation. The permutation invariant (symmetric) down-sampling method like max / mean / sum-pooling, or even sophisticated pooling method like [30, 31] can be used for the aggregation function in the readout layer. In this work, we empirically selected mean-pooling method for global graph pooling. The element-wise mean operation of node embeddings of the last layer *K* of GCN as:

$$\begin{array}{c} h_{S^{\left(t\right)}}=MEAN\left(H_{K}^{\left(t\right)}\right) \end{array}$$ (6)

for the global graph snapshot embedding at *t* ∈ {1, 2, …, *T*}; *h*_*S*^(*t*)^_.

### 4.4 Attention mechanism

To apprehend the dynamic (temporal) information of graph snapshots, we use attention mechanisms. We adopt two well-known attention mechanisms: additive attention [35] and scaled dot-product attention [36]. From the graph snapshot embeddings $h_{s}=\{h_{s^{\left(1\right)}},h_{s^{\left(2\right)}},\cdots ,h_{s^{\left(T\right)}}\}$, the goal is to learn the attention weights and use them to aggregate the weighted inputs.

Introduced in [20, 35], for the additive attention, we retrieve the context vector *m*_*s*_ by applying element-wise mean operation of embeddings of *h*_*s*_. The context vector *m*_*s*_ is used as a query (Q) of the attention mechanism and *h*_*s*_ is used for the key (K) and value (V). For the additive attention, query and key are concatenated and fed to a feed-forward neural network to produce the attention scores *z*. Attention weights are calculated as:

$$\begin{array}{c} Attention(Q,K,V)=Softmax(MLP(Q;K))V\\ Softmax(z_{i})=\frac{e^{z_{i}}}{\sum_{j=1}^{K}e^{z_{j}}},z=(z_{1},z_{2},\cdots ,z_{K})\in {\mathbb{R}}^{K} \end{array}$$ (7)

Scaled dot-product attention consider the dot-product similarity of the embeddings when calculating the attention scores. We adopt self-attention which the query (Q), key (K), value (V) is all $h_{s}=\{h_{s^{\left(1\right)}},h_{s^{\left(2\right)}},\cdots ,h_{s^{\left(T\right)}}\}$ as:

$$Attention(Q,K,V)=Softmax(\frac{\left(QK^{T}\right)}{\sqrt{d_{k}}})V$$ (8)

The softmax result of normalized similarity measures of snapshots is applied to calculate the attention weights for the *h*_*s*_ where *d*_*k*_ is the dimension of *h*_*s*^(*t*)^_.

The outputs of the two different attention layers are both the weighted sequences of the snapshot embeddings. The element-wise average of the *T* snapshots where attention weights are applied is used to retrieve the global graph embedding *h*_*G*_ as:

$$\begin{array}{c} h_{G}=\frac{1}{T}\sum_{i=1}^{T}h_{S^{\left(i\right)}} \end{array}$$ (9)

### 4.5 Training & prediction

For the graph classification task, the graph embedding *h*_*G*_ is fed to the multi-layer perception as:

$$\begin{array}{c} \hat{y}=Softmax\left(MLP\left(h_{G}\right)\right) \end{array}$$ (10)

The $\hat{y}\in R^{\left|class\right|}$ is the probabilities of veracity labels where *class* = {*T*, *F*, *U*, *N*} or *class* = {*R*, *N*}.

Our supervised graph classification model is trained with the cross-entropy loss of the predictions and ground truth labels. The loss function of our model is defend as:

$$\begin{array}{c} L=\sum_{i=1}^{\left|class\right|}-y_{i}^{T}log{\hat{y}}_{i} \end{array}$$ (11)

where *y*_*i*_ is the ground truth label for the claim *c*_*i*_.

## 5 Experiments

In this section, we perform experiments on three real-world datasets and compare the performance of the proposed model, *Dynamic GCN*, with other rumor detection baselines. Furthermore, we conduct ablation studies and analyze the results on different snapshot counts and variants of the sequential learning methods.

### 5.1 Datasets

We evaluate the proposed model with three publicly available rumor detection datasets: *Twitter15* [13], *Twitter16* [16], and *Weibo* [18]. These datasets contain rumor propagation trees, where nodes are posts and links are responsive relations such as replies or retweets, with one of the four ground truth veracity labels (True rumor, False rumor, Unverified rumor, Non-rumor) for *Twitter15* and *Twitter16* and two classes (Rumor, Non-rumor) for *Weibo* dataset. The detailed statistics of the datasets are provided in Table 1. We used the bag-of-words (BoW) features by selecting the top 5,000 vocabularies for the corpus by *TF-IDF*; thus, each post initially contains 5,000 features.

**Table 1:** The statistics of the rumor detection datasets.

	*Twitter15*	*Twitter16*	*Weibo*
# of root posts	1490	818	4664
# of users	276,663	173,487	2,746,818
# of posts	331,612	204,820	3,805,656
# of true rumors	372	205	0
# of false rumors	370	205	2313
# of unverified rumors	374	203	0
# of non-rumors	374	205	2351
avg. max. time (hours)	1,337	848	2,461
avg. # of posts / event	222.6	250.4	816.0
max. # of posts / event	1,768	2,765	59,318

### 5.2 Baselines

We compare our Dynamic GCN model with the following rumor detection baseline models:

- DTC [7]: A decision tree-based classifier with handcrafted features to identify the credibility of microblog posts related to trending topics.
- RFC [11]: A random forest based-ranking method that elaborates the inquiry phrases of posts.
- SVM-TS [12]: An SVM model that captures the temporal characteristics of social context features of posts.
- SVM-TK [16]: An SVM model with a tree kernel that captures higher-order patterns of propagation structures of rumors.
- GRU [18]: An RNN-based model that learns contextual information from continuous representations of relevant posts over time.
- RvNN [21]: A recursive neural network-based model which captures the structural patterns of a top-down and bottom-up rumor propagation trees.
- Bi-GCN [26]: A graph convolutional network-based model, which captures propagation patterns with message passing architecture.
- DynGCN (Proposed): A graph convolutional network-based model with attention mechanisms to capture temporal dynamics of graph snapshots.

We haven’t included the Propagation Path Classification (PPC) model [20] and Global-Local Attention Network (GLAN) model [27] as our baselines since both methods include crawled user profiles as additional input features (such as whether the user is suspended or verified), which could be too biased at the time of current work. A few years had passed since the initial collection of the datasets, the results could be distorted and might be too much depended on when the user profiles were crawled. Instead, we compare our model with the state-of-the-art model [26], which considers the posts relations without additional crawled user profiles.

### 5.3 Experimental setup

We conducted 10 runs of 5-fold cross-validation and reported the average accuracies and F1 scores by each label. For the fair comparison, for the models with early stopping method [50] such as Bi-GCN and ours, we randomly splitted 4-fold of training set into 80% training set and 20% validation set, which eventually making 16:4:5 splits for train, validation, and test sets. The validation set was used for early stopping with patience of 10 epochs.

The model has 256 hidden dimensions for a single graph snapshot, including root feature enhancement and bi-directional representation. We set 2-layer GCNs and used rectified linear units for the non-linearity. We adopt the dropout [51] rate of 0.5 for GCN layers and DropEdge [52], graph data augmentation method, rate with 0.2. We train our model with Adam optimization algorithm [53] with the initial learning rate 5E-4 and a maximum of 200 epochs if not early stopped.

Our model is implemented in PyTorch [54] with PyTorch Geometric [55] for the message passing framework. For the baseline models, we conduct experiments with the authors’ provided codes with the same hyperparameters that were reported, respectively. For the fair comparison, we directly cited (*) some of the metrics already reported in original papers [16, 18] with equivalent experimental settings due to some handcrafted features that are unavailable at the time of the reproduction.

### 5.4 Performance evaluations

Tables 2 and 3 summarize the overall performances of the rumor detection task of the proposed model with other baselines. The reported performances are accuracies and F1 scores of DynGCN with both additive attention (ADD) and dot-product attention (DOT) with the sequential (S) snapshots or temporal (T) snapshots size of 3. The accuracy results of DynGCN with additive attention shows (S) 0.818, (T) 0.827 in *Twitter15*, and (S) 0.828, (T) 0.836 in *Twitter16*. The accuracy results of DynGCN with dot-product attention shows (S) 0.819, (T) 0.821 in *Twitter15*, and (S) 0.829, (T) 0.824 in *Twitter16*. Although the two attention methods don’t show significant performance differences, the model with additive attention and temporal snapshots outperformed others. Furthermore, both variants of attention models outperform other state-of-the-art models, such as Bi-GCN and RvNN, in both *Twitter15* and *Twitter16* with the aid of evolving patterns. The results indicate that taking account of the temporal information and evolving pattern of rumor propagation is beneficial. A similar result is shown for the binary classification task of the *Weibo* dataset. Although the timestamps aren’t retrieval for *Weibo*, the experiment with the sequential snapshots with the snapshot size of 3 shows the improved performance.

**Table 2:** Overall performance of rumor detection task on *Twitter15* and *Twitter16*.

Model		*Twitter 15*					*Twitter 16*				
		Accuracy	TR	FR	UN	NR	Accuracy	TR	FR	UN	NR
			F1	F1	F1	F1		F1	F1	F1	F1
DTC [7]*		0.454	0.317	0.355	0.415	0.733	0.465	0.419	0.393	0.403	0.643
RFC [11]*		0.565	0.401	0.422	0.543	0.810	0.585	0.547	0.415	0.563	0.752
SVM-TS [12]*		0.544	0.404	0.472	0.483	0.796	0.574	0.571	0.420	0.526	0.755
SVM-TK [16]*		0.667	0.772	0.669	0.645	0.619	0.662	0.783	0.623	0.655	0.643
GRU [18]		0.641	0.688	0.634	0.571	0.684	0.633	0.577	0.715	0.527	0.617
RvNN [21]		0.723	0.821	0.758	0.654	0.682	0.737	0.835	0.743	0.708	0.662
Bi-GCN [26]		0.814	0.793	0.811	0.872	0.768	0.804	0.718	0.787	0.799	0.895
DynGCN w/additive attention	Sequential	0.818	0.860	0.793	0.761	0.779	0.828	0.765	0.736	0.826	0.637
	Temporal	**0.827**	0.837	0.769	0.820	0.746	**0.836**	0.880	0.804	0.853	0.741
DynGCN w/dot-product attention	Sequential	0.819	0.871	0.816	0.771	0.800	0.829	0.873	0.756	0.775	0.708
	Temporal	0.821	0.859	0.806	0.765	0.782	0.824	0.876	0.848	0.767	0.742

**Table 3:** Overall performance of rumor detection task on *Weibo*.

Model	*Weibo*		
	Accuracy	Rumor	Non-rumor
		F1	F1
DTC [7] *	0.831	0.831	0.819
RFC [11] *	0.849	0.864	0.830
SVM-TS [12] *	0.857	0.861	0.857
GRU [18]	0.910	0.914	0.906
RvNN [21]	0.908	0.905	0.911
Bi-GCN [26]	0.928	0.928	0.928
DynGCN w/ ADD (Seq)	**0.936**	0.936	0.936
DynGCN w/ DOT (Seq)	0.932	0.932	0.932

It is demonstrated that the traditional machine learning-based methods with handcrafted features, (DTC, RFC, SVM-TS, SVM-TK), show lower performances compare to other deep learning-based methods (GRU, RvNN, BiGCN, DynGCN). However, SVM-TS and SVM-TK show superior results within the traditional handcrafted methods since these models are able to utilize temporal features. It is constructive to consider temporal information of rumor for rumor detection.

Finally, among the propagation-based baselines, a graph-based models, DynGCN and Bi-GCN, outperforms other baselines such as RvNN or GRU since graph convolutional network can better capture the structural representation of rumor diffusion.

### 5.5 Ablation study

In order to see the performance of our model in different settings, we report the following ablation studies. The performance results with different snapshot counts for sequential and temporal snapshots, with different learning algorithms for combining snapshot sequences, and attention weights of additive attention and dot-product attention.

#### 5.5.1 Different snapshot counts

**Fig 4:**
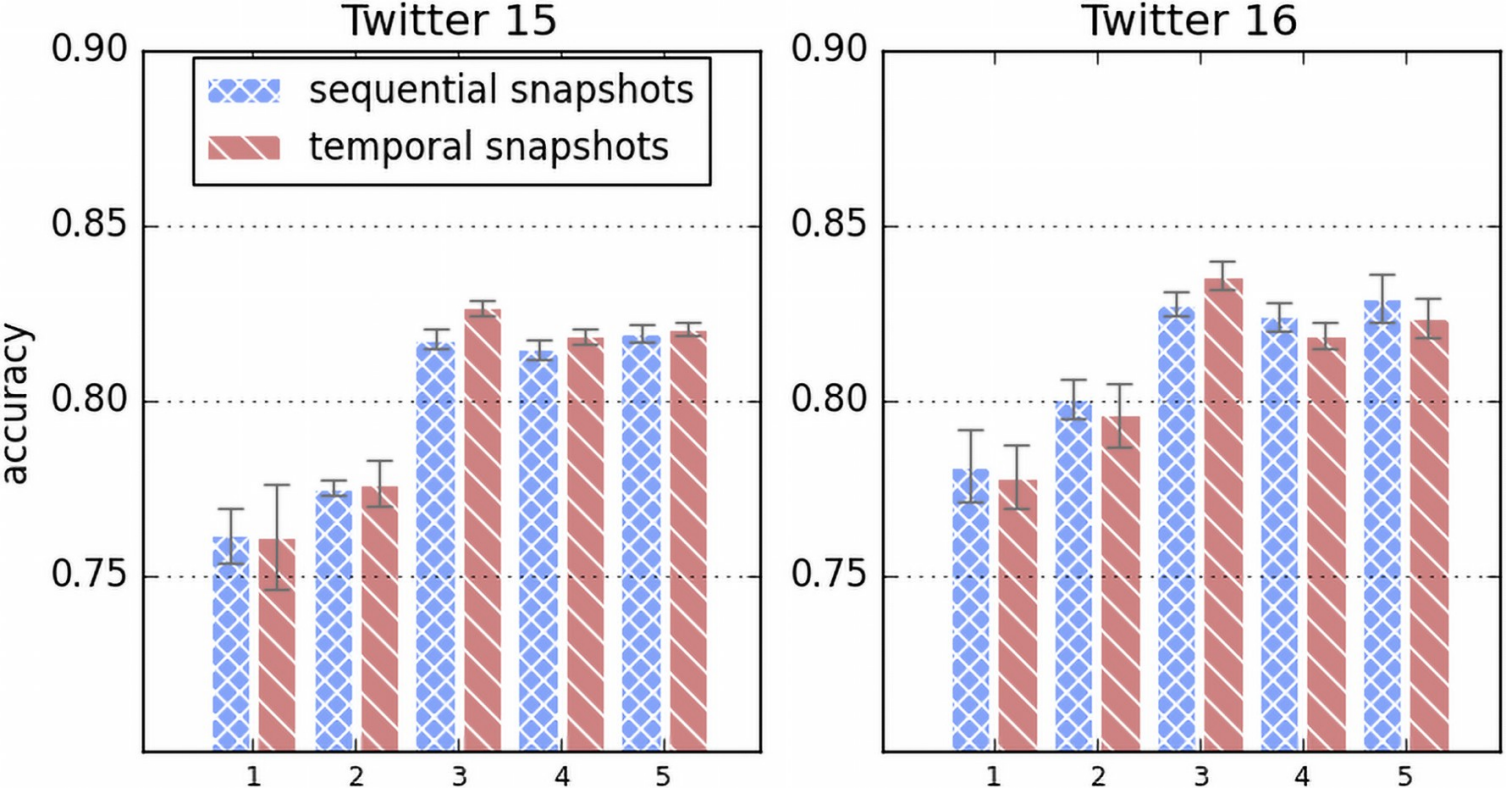
Difference in accuracy for different snapshot counts.

Fig 4 is the result of DynGCN with the snapshot counts of 1, 2, 3, 4, and 5 with dot-product attention. Although there aren’t significant correlations in the aspect of accuracy with the counts, adopting multiple snapshots shows better performance compare to a single static snapshot in both sequential and temporal snapshots. However, we observed that simply applying larger snapshot counts won’t produce a performance improvement and believe this can be a hyperparameter for the dataset.

#### 5.5.2 Different learning methods for the sequence

**Fig 5:**
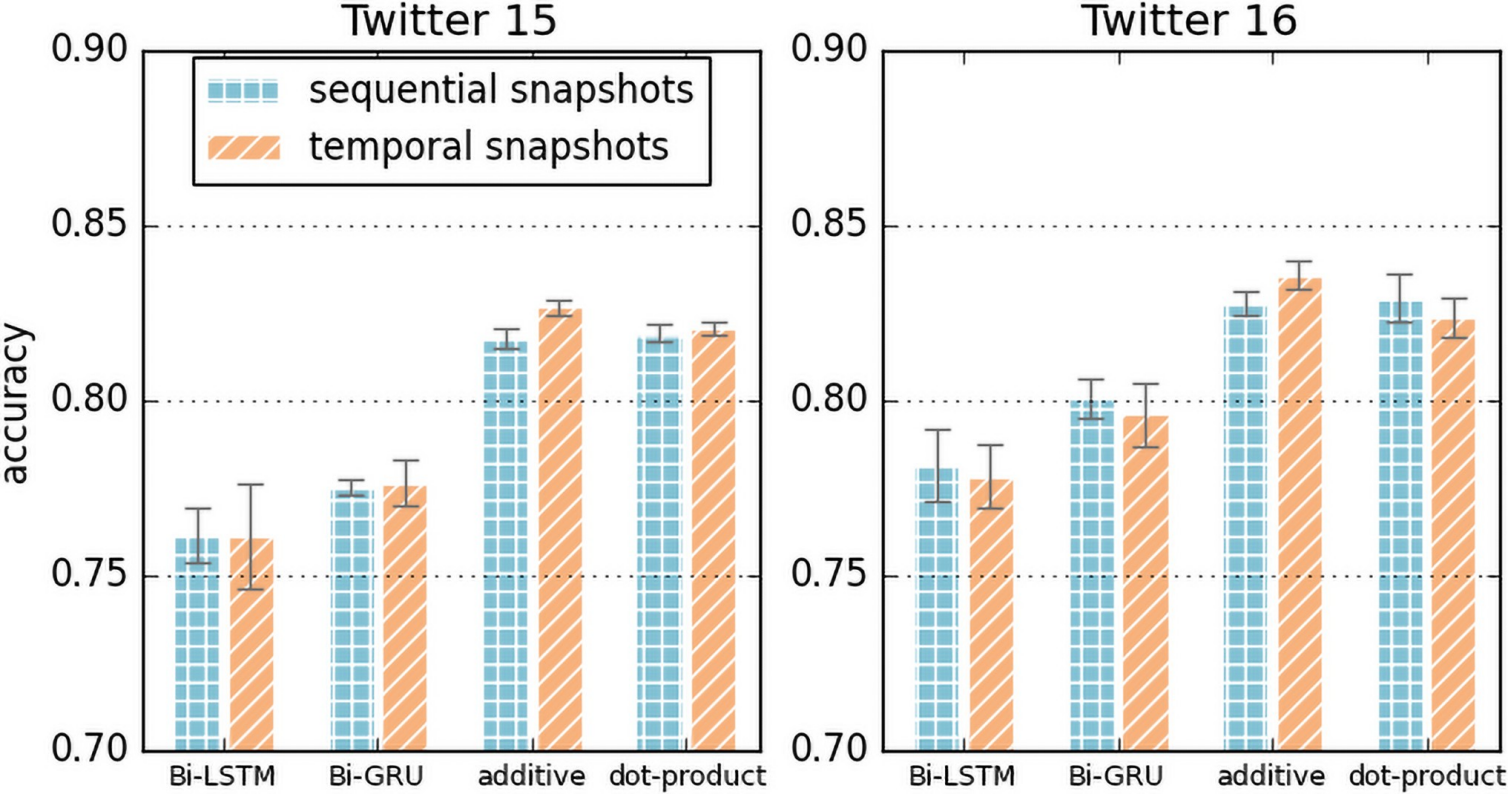
Differences in accuracy of various sequence learning methods.

The attention layer of our model can be replaced with other Seq2Seq [34] models since the inputs to the attention layer are a sequence of snapshot representations. Fig 5 is the result of different sequence learning methods (Bi-LSTM, Bi-GRU, additive attention, and dot-product attention (self-attention)) with the snapshots count of 3. Attention mechanisms that are used for a weighted sum of sequential and temporal snapshot representations outperform the other RNN-based models. Bidirectional LSTM/GRU show low performance in aggregating the temporal representation of graph snapshots. We suspect the results of the relatively low performance of Bi-LSTM and Bi-GRU are due to the short sequence of global graph snapshots. [34]

#### 5.5.3 Effects of the attention mechanisms

**Fig 6:**
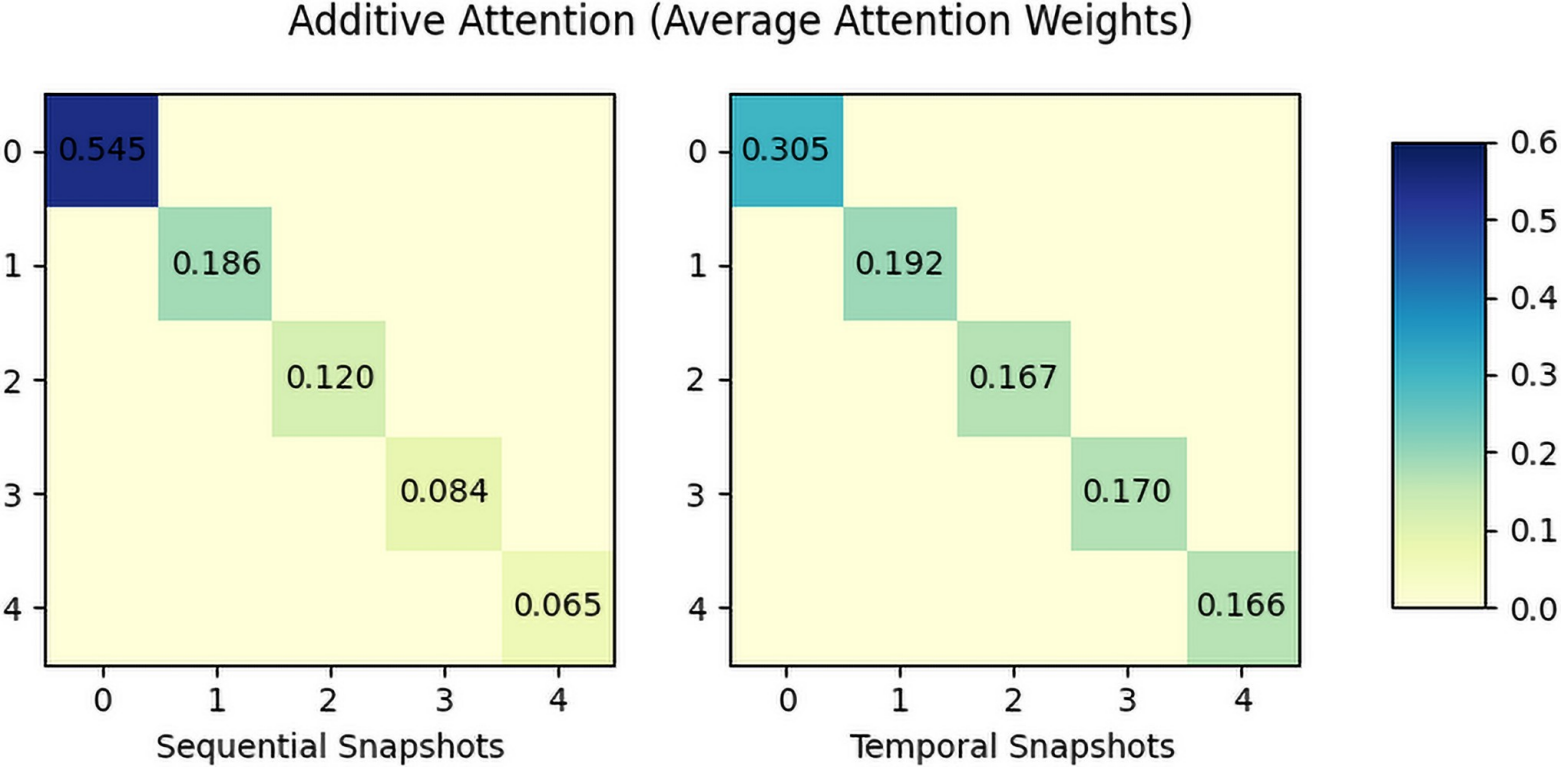
Average attention weights of additive attention on *Twitter16*.

**Fig 7:**
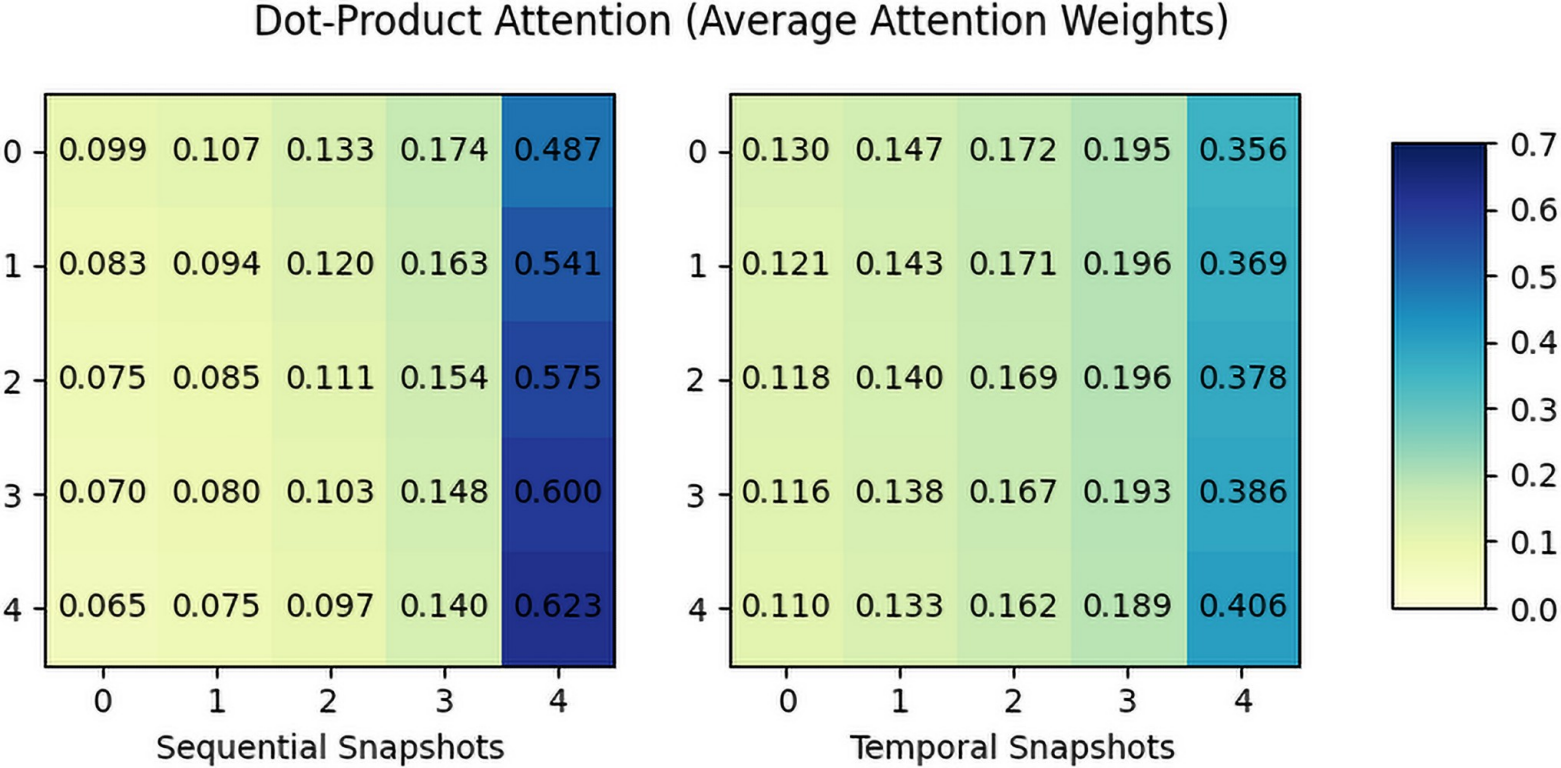
Average attention weights of dot-product attention on *Twitter16*.

Finally, Figs 6 and 7 are the visualization of the average attention weights of additive attention and dot-product attention. Notice that additive attention takes a context vector as a query for the attention; thus, the attention matrix is diagonal, while dot-product attention is a self-attention. The result shows the additive attention considers the early stage of the rumor propagation while the dot-product attention significantly considers the snapshots in the end-stage. This can be interpreted as that the additive attention reply on the context query to understand the global or overall propagation while dot-product attention relies on the input sequence to jointly understand the overall pattern. Although the weight itself depends on the dataset, we could see that each attention mechanism represents the propagation structure in its own way.

## 6 Conclusion

In this research, we propose Dynamic GCN, an end-to-end GCN-based model with attention mechanisms, for rumor detection. The model is able to capture the dynamics of rumor propagations using sequential snapshots and temporal snapshots. We empirically evaluate our model with three real-world datasets and compare the performance of the rumor detection (veracity classification) task with other rumor detection baselines. The results show that our model outperforms other state-of-the-art methods. The ablation studies report performance differences with snapshots counts, learning sequence variants, and the weights for the different attention mechanisms. We believe there is still room for improvement in the context of GCNs variants, global graph pooling, and additional features from different contexts.
